# Cardiac myxosarcoma: A case report

**DOI:** 10.22088/cjim.12.2.228

**Published:** 2021-03

**Authors:** Manouchehr hekmat, Alireza Omidi Farzin, Zahra Ansari Aval, Kamal Fani, Azadeh Heidarpour

**Affiliations:** 1 Department of Cardiac Surgery, Modarres Hospital, School of Medicine, Shahid Beheshti University of Medical Sciences, Tehran, Iran; 2 Department of Cardiac Anesthesia, Modarres Hospital, School of Medicine, Shahid Beheshti University of Medical Sciences, Tehran, Iran; 3 Department of Obstetrics and Gynecology, Tehran University of Medical Sciences, Tehran, Iran

**Keywords:** Myxosarcoma, Transthoracic Echocardiogram (TTE)

## Abstract

**Background:**

It is a rare cardiac malignant primary tumor that seems to derive from the same cellular line as myxomas, but the prognosis is very different. It is a rare cardiac malignant primary tumor that seems to derive from the same cellular line as myxomas, but the prognosis is very different. It is a rare cardiac malignant primary tumor that seems to derive from the same cellular line as myxomas, but the prognosis is very different. Cardiac myxosarcoma is a rare neoplasm that appears to rise from the same cellular source like myxoma. It is difficult to differentiate a myxoma tumor from a myxosarcoma tumor because of its appearance and pathology examination. Myxosercoma tumor requires surgery and chemoradiotherapy, but myxoma is treated only by surgery.

**Case Presentation:**

We describe a case of a 58-year-old patient with a left atrium myxosarcoma, presenting with congestive heart failure. Transthoracic echocardiogram (TTE) showed a large polypoid and mobile mass in the left atrium, the patient underwent cardiac surgery and the tumor was successfully extracted, and histopathological result revealed typical features of myxoma. 15 days after surgery, he underwent explorative laparatomy because of progressive GI bleeding. Laparatomy revealed extensive metastatic masses in abdomen and the pathology diagnoses was myxosaroma. Unfortunately, in spite of supportive care, the patient expired on postoperative day one.

**Conclusion:**

It is difficult to differentiate a myxoma tumor from a myxosarcoma tumor because of its appearance and pathology examination. Maybe magnetic resonance imaging can help us to achieve more data suggesting malignancy.

Primary cardiac tumors are extremely uncommon, with incidence of 0.001 to 0.3% (1, 2). Most of these tumors (~75%) are benign; and atrial myxoma is the most common cardiac tumor. Myxosarcoma is a rare malignancy and as it has a similar histological appearance to myxoma, differentiation between them is very difficult (3). Myxosarcomas are locally invasive and they have a poor prognosis compared with myxoma. Myxosarcoma mostly originate from the left atrium (4-6), but it can also originate in other sites (7-9). Cardiac sarcomas usually appeared with mysterious symptoms in young and middle aged patients. They usually have a poor prognosis with overall survival ranging from 6 to 12 months (10, 11). Symptoms associated with cardiac sarcomas are various and include: pericardial effusions with tamponade, congestive heart failure, dyspnea, chest pain, syncope, sudden cardiac arrest, fever and weight loss (12-14). After institutional ethics committee approval (IR.SBMU.RETECH.REC.1399.658), we report a patient with a myxosarcoma with extensive metastatic masses.

## Case presentation:

**Fig 1:**
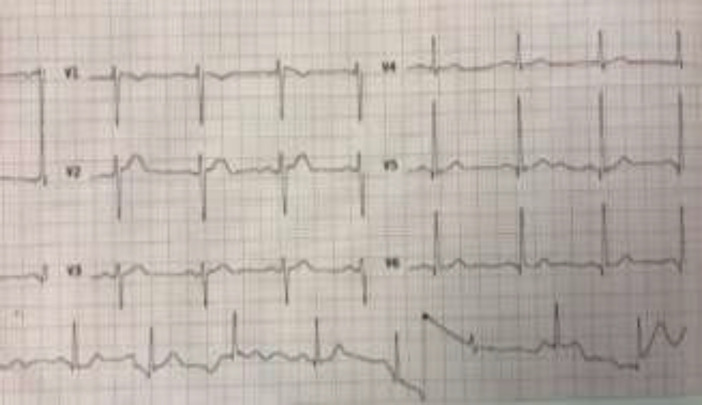
ECG of the patient with Myxosarcoma

A 58-year-old man was admitted to our hospital who complained of progressive exertional dyspnea for one month, chest pain and lower GI bleeding in November 2018.The patient had past medical history of hypertension and GI bleeding. His vital signs were stable. On physical exam, conjunctiva was pale. Lungs auscultation was clear. Cardiovascular exam showed tumor plop sound at the apex. Abdominal exam was normal. Laboratory findings were as follows: WBC 11.3 K/ul, Hb 7.9g/dl, Hct 23.8 %, platelets 500K/ul. Electrolyte panel findings were as follows: Na 141 mmol/L, K 4.0 mmol/L, Urea 35 mg/dl, Cr 1.27 mg/dl. ECG showed sinus rhythm (fig 1).

**Fig 2:**
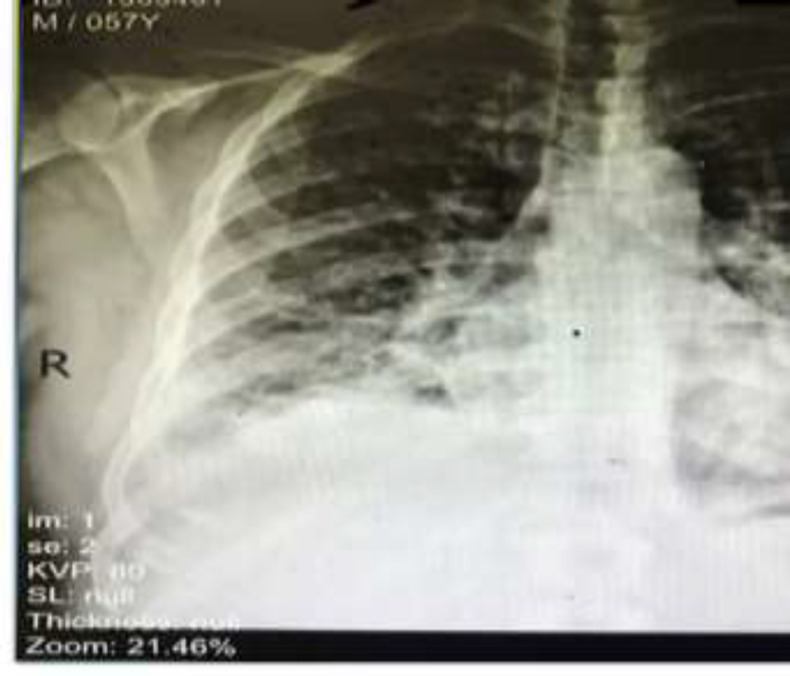
Chest x-ray of the patient with Myxosarcoma

**Figure 3:**
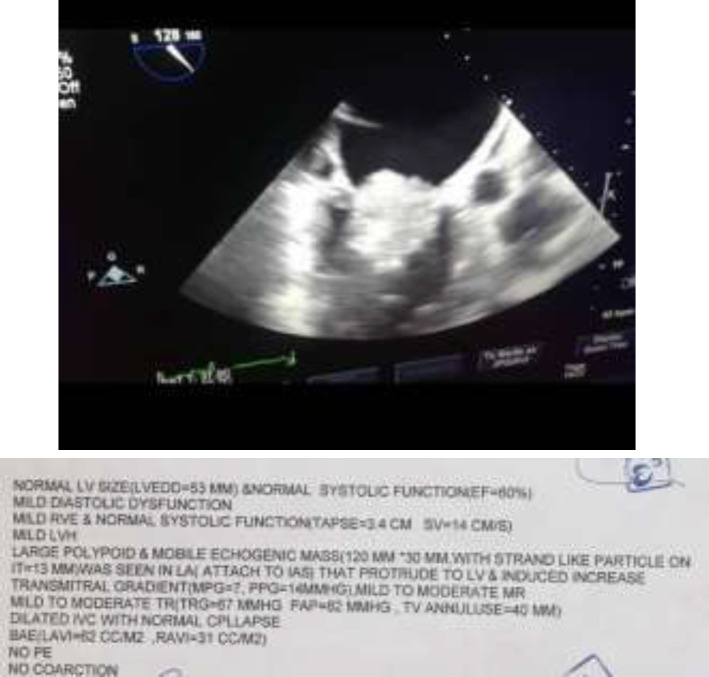
Echocardiography of the patient with Myxosarcoma

Transthoracic echocardiogram (TTE) revealed EF 60%, mild mitral regurgitation and a large polypoid and mobile tumor (120 mm*30 mm with strand like particle on it) in the left atrium (fig 3).

**Figure 4:**
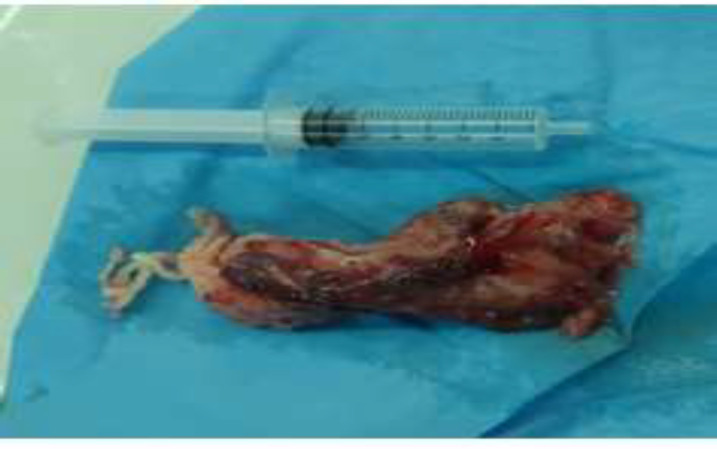
Atrium mass in the patient with Myxosarcoma

The patient underwent median standard sternotomy and exploration. A large, 10 cm × 4 cm mass was located in left atrium, attached via to the pulmonary vein. The mass was extracted, and histopathological result revealed typical features of myxoma (fig 4).

**Figure 5.**
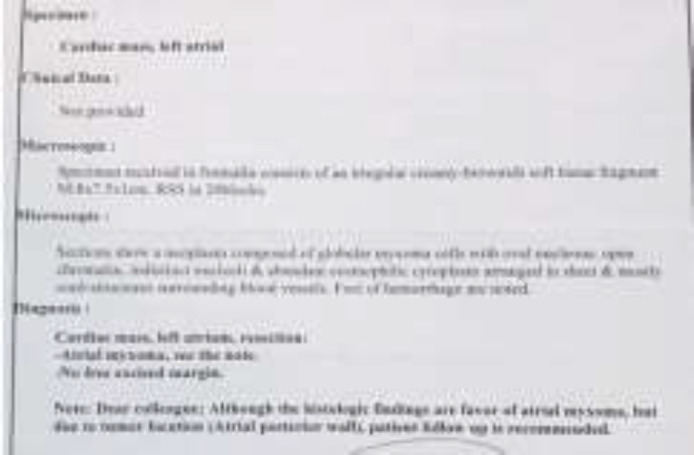
Pathology report of the patient with Myxosarcoma

**Figure 6:**
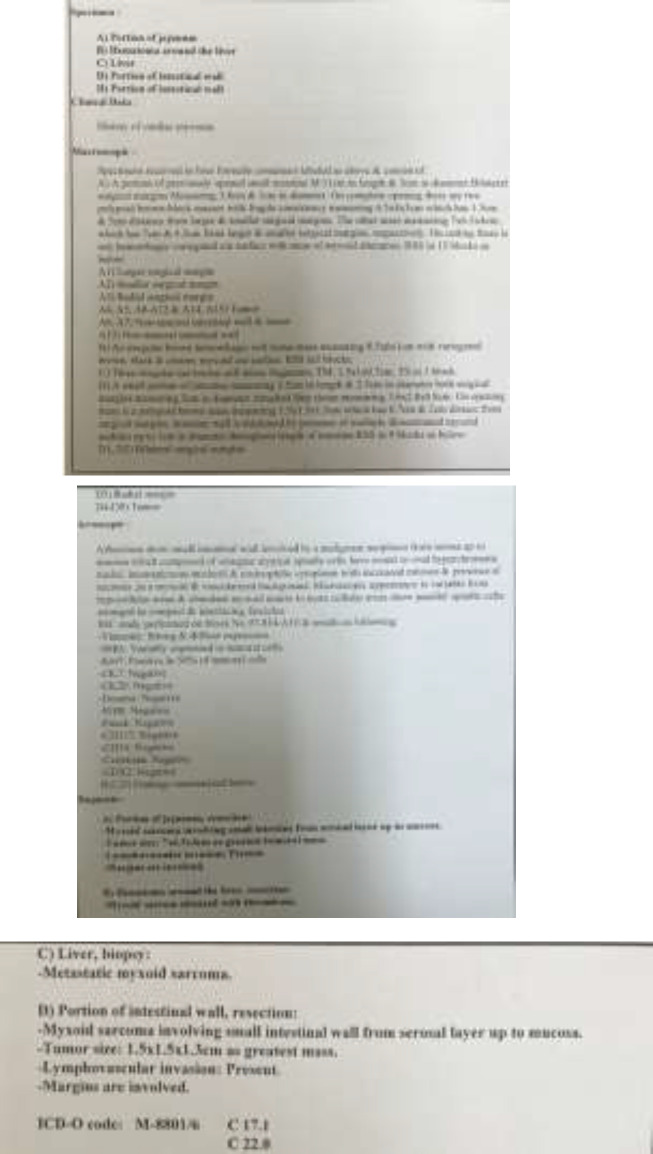
Pathology report of the patient with Myxosarcoma

After the operation, the patient was sent to intensive care unit. After ten days, the patient was discharged from the hospital. The control post operation echocardiography was normal.15 days later, the patient came to the emergency room with pleuritic chest pain, illness, fatigue and anemia (Hb=7.7). TEE disclosed moderate MR (mitral regurgitation) and no pericardial effusion. Endoscopy showed small size sliding hiatal hernia and gastritis. However, the treatment for GI signs did not improve the patient's complaints. Abdominal pain and vomiting developed gradually. And GI bleeding occurred again. The patient underwent endoscopy and colonoscopy that was suspicious to obstruction. He underwent exploratory median laparatomy. Opening the abdomen, we could see extensive metastatic masses in liver and invagination was found because of a 5 cm mass, 10 cm to ligamentum teres. The pathology diagnosis was myxosaroma involving small intestine from serosal layer up to mucosa, lymphovascular invasion was present and margins were involved (figs. 5, 6).

**Figure 7:**
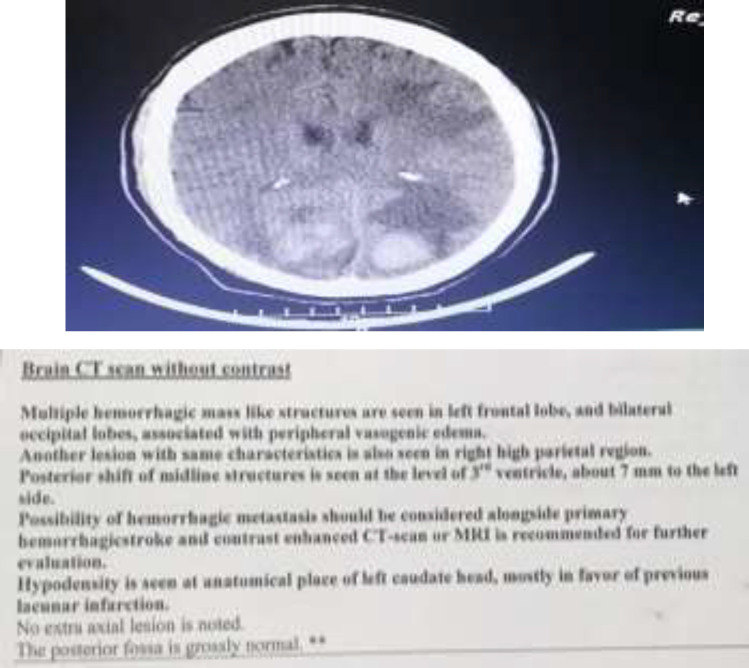
Brain CT of the patient with Myxosarcoma

The mass and metastases specimen were removed and sent to pathology. Loss of consciousness, headache and blurred vision occurred 5 hours after surgery. The patient underwent brain scan computed tomography (CT) (fig. 7).

Brain CT Scan showed multiple hemorrhagic mass like structure are seen in left frontal lobe and bilateral occipital lobes. Posterior shift of midline structures is seen at the level of third ventricle, about 1mm to the left side Possibility of hemorrhagic metastasis should be considered alongside primary hemorrhagic stroke. Unfortunately, in spite of supportive care the patient expired on postoperative day one.

## Discussion

This case illustrates an uncommon, malignant case of a left atrial myxosarcoma with rapid progression of symptoms which proved fatal. Myxosarcoma is an uncommon type of primary cardiac malignant tumor (1-3). Only half of cases can be treated by resection, but the local recurrence is most common cause of mortality (50%). In spite of combination of radiation, chemotherapy with or without surgery but they do not prevent local recurrences and metastasis. The recurrence makes the prognosis poor with a mean survival time of about 12 months after diagnosis (15-17). It is difficult to differentiate a myxoma tumor from a myxosarcoma tumor because of its appearance and pathology examination. Maybe magnetic resonance imaging can help us achieve more data suggesting malignancy.
